# Understanding the Cognitive Load of Cell Phone Use While Walking: Distinct Effects of Texting and Phone Conversation

**DOI:** 10.3390/brainsci16080807

**Published:** 2026-07-30

**Authors:** Patrícia de Morais Ferreira Brandão, Heloisa Helena Batista Ferreira, Giovanna Souza Oliveira, Sidney Afonso Sobrinho Junior, Gustavo Christofoletti

**Affiliations:** Faculty of Medicine, Institute of Health, Federal University of Mato Grosso do Sul, UFMS, Campo Grande 79060900, Brazil; patricia.morais@ufms.br (P.d.M.F.B.); heloisa.h@ufms.br (H.H.B.F.); s.giovanna@ufms.br (G.S.O.); junioorsobrinho@gmail.com (S.A.S.J.)

**Keywords:** aging, cognition, executive function, cognitive load, dual task performance, walking, cell phone, motor control, accidental falls

## Abstract

**Highlights:**

**What are the main findings?**

- Older adults experience greater cognitive–motor interference than young adults when performing the dual task of walking while using a cell phone.
- In a cognitively preserved sample, age was the strongest determinant of mobility performance during the dual task, explaining 42.4% of the variance in gait. Cognition accounted for 16.6% of gait variance.

**What are the implications of the main findings?**

- Increased cognitive–motor interference during cell phone dual-tasking may potentiate age-related changes that compromise mobility and safe walking.
- Understanding how different cell phone-related tasks affect cognitive–motor performance may help guide interventions designed to preserve mobility and reduce functional decline associated with aging.

**Abstract:**

**Background**: Aging is marked by a series of changes that impair mobility. These changes are accompanied by declines in balance, postural control, and cognitive function. While the relationship between balance, postural control, and mobility has been extensively investigated, evidence on how different cognitive demands influence mobility performance in daily life remains limited. Accordingly, this study investigated how different cognitive demands associated with cell phone use affect mobility performance in young and older adults. **Methods**: In this cross-sectional study, 124 cognitively preserved participants (*n* = 62 young and *n* = 62 older adults) were enrolled. The participants were subjected to three walking conditions: without distraction, while texting, and while talking on the phone. Wearable motion sensors were used to quantify mobility performance across different task-specific components. Age and cognitive function were assessed to investigate their roles in mobility performance. **Results**: Older adults exhibited poorer gait performance than young adults, demonstrating longer completion times across multiple functional mobility tasks. Texting and phone conversations adversely affected gait performance to a similar extent. Age explained up to 42.4% of the variability in mobility performance during dual task, whereas cognitive function accounted for an additional 16.6% of the variance. **Conclusions**: In a cognitively preserved sample, the negative impact of cell phone use while walking was greater in older than in young adults. Age was the primary determinant of mobility performance while cognition played a lesser role. These findings support the need for interventions addressing age-related changes and cognitive demands to improve mobility in older adults.

## 1. Introduction

The brain is one of the most complex organs in the human body. Composed of billions of neurons organized through extensive neural networks, the brain is responsible for integrating sensory information, supporting cognitive processes, and generating motor responses [1,2]. Through continuous communication among cortical and subcortical structures, the brain enables perception, attention, memory, decision-making, and movement control [3,4].

Cognitive and motor processing begins with the detection of environmental stimuli by specific sensory receptors. These receptors convert physical and chemical stimuli into neural signals that are transmitted to the central nervous system (CNS) [5]. The visual, auditory, vestibular, somatosensory, proprioceptive, olfactory, and gustatory systems provide complementary information regarding the external environment and the internal state of the body [6]. The success of motor performance relies on efficient interactions between sensory processing and cognitive functions. Executive functions, working memory, attention, concentration, and information processing are necessary for selecting relevant stimuli, allocating attentional resources, and coordinating motor actions [7,8,9]. To ensure safe and effective movement during daily activities, the brain constantly integrates sensory and cognitive information, which is particularly detrimental when individuals are required to perform simultaneous tasks [10,11].

From a physiological perspective, sensory stimuli are first detected by specialized receptors in the peripheral nervous system and then transmitted to the CNS through afferent pathways [12]. In the brain, sensory information is processed in the primary sensory areas and subsequently interpreted within the association cortices, enabling stimulus recognition and contextual understanding [13,14]. Depending on the nature and complexity of the task, neural processing may trigger rapid sensorimotor reflexes or engage the cognitive network. The cortical motor areas coordinate the selection and execution of appropriate motor responses in conjunction with the basal ganglia and cerebellum, which contribute to movement planning, timing, coordination, and adaptation [15,16,17]. The integration of neural mechanisms provides the basis for motor control.

Motor control emerges from the coordinated interplay of several neural circuits. It arises from the continuous integration of sensory, cognitive, and motor processes, allowing individuals to perceive, interpret, and respond to environmental demands. These mechanisms emerge early in life and contribute to motor learning [15,18,19]. Throughout life, experience-dependent neuroplasticity facilitates this process by the maturation and reorganization of neural networks that support sensorimotor integration and adaptive motor behavior [20,21]. As motor and cognitive systems mature, sensory information is integrated, enabling individuals to adapt the motor control to environmental conditions and task demands. However, while early life is characterized by the acquisition and refinement of motor skills through neuroplasticity, aging is often accompanied by a progressive decline in the neural systems that support cognition and motor performance, therefore affecting motor control [22].

Among all the aspects of motor control influenced by aging, walking holds particular importance because of its role in preserving independence in daily activities. Walking is no longer considered an automatic activity. Instead, it is recognized as a complex task that requires continuous interaction between the cognitive and motor systems. In this scenario, dual tasks have been employed to examine the interactions between cognition and walking. Given that gait and cognition share overlapping neural networks, heightened cognitive demands can diminish the attentional resources available for gait regulation, potentially impairing mobility [22,23].

The cognitive and motor interactions become relevant in the context of aging. Age-related decline is often associated with impairments in balance, gait, postural control, reaction time, and motor adaptation, reducing the ability to respond efficiently to environmental challenges [24]. Previous studies confirm that older adults become particularly vulnerable to cognitive–motor interference during everyday activities that require the simultaneous performance of motor and cognitive tasks, making them especially vulnerable to dual-task activities [25,26,27]. This is particularly concerning, as it may compromise the health and quality of life of older adults.

Promoting healthy aging and preserving quality of life have become a major public health challenge worldwide. Although increased life expectancy represents an important achievement, longevity is frequently accompanied by functional decline and a high prevalence of chronic diseases and physical disabilities. These aspects may compromise mobility, functional independence, social participation, and overall well-being, leading to increased healthcare utilization and economic burden [28,29]. Accordingly, several preventive interventions have been developed to preserve the functional capacity and mobility of older adults. In recent years, particular attention has been directed toward dual-task paradigms that investigate the effects of performing two tasks simultaneously [30,31,32]. These studies have provided valuable insights into the challenges imposed by aging, demonstrating that the concurrent execution of motor and cognitive tasks increases attentional demands and may negatively affect motor performance. This aspect has become increasingly relevant with the widespread use of cell phones, which frequently require users to divide their attention while performing motor tasks such as walking [33,34].

The growing interest in dual-task paradigms is attributed to the close interaction between cognition and motor control. When cognitive demands increase, fewer attentional resources remain available for gait regulation, potentially impairing mobility performance. The use of cell phones in dual-task conditions has introduced additional mobility challenges for older adults, as activities such as texting and talking on the phone while walking have become common in daily life [34].

Texting and talking on the phone represent different levels of cognitive demand while performing the same motor task. This makes cell phone use an ecologically valid model for investigating how cognitive processing influences mobility across the lifespan. Understanding how older adults respond to dual-task demands is important for developing targeted interventions to maintain independence and reduce the risk of falls in this population. In this context, this study investigated the effects of cell phone use while walking on gait performance in older and young adults, comparing the impact of motor and cognitive dual-task demands on mobility-related outcomes in different age groups.

## 2. Methods

This cross-sectional study included 124 participants: 62 older and 62 young adults. G*Power software (version 3.1.9.7; Heinrich-Heine-Universität, Düsseldorf, Germany) was used to calculate the sample size. The calculation was performed for an *F* test using within–between interaction, considering two groups and three walking conditions. The parameters adopted were an effect size of *f* = 0.15, α = 0.05, statistical power of 95%, correlation among repeated measures of 0.50, and nonsphericity correction of 1.0. The estimated minimum sample size was 116 participants, with 58 participants in each group. To maintain balanced groups and account for possible data loss, 124 participants were enrolled, 62 in each group.

All the participants were recruited from the community. The methodological protocol of this study was approved by the Research Ethics Committee of the Federal University of Mato Grosso do Sul (approval no. # 7.465.300, date of approval: 25 March 2025). The methodological procedures were conducted in accordance with the Declaration of Helsinki and its amendments. All participants provided written informed consent prior to data collection. Participants were reimbursed for expenses related to their transportation to the assessment location.

**Figure 1:**
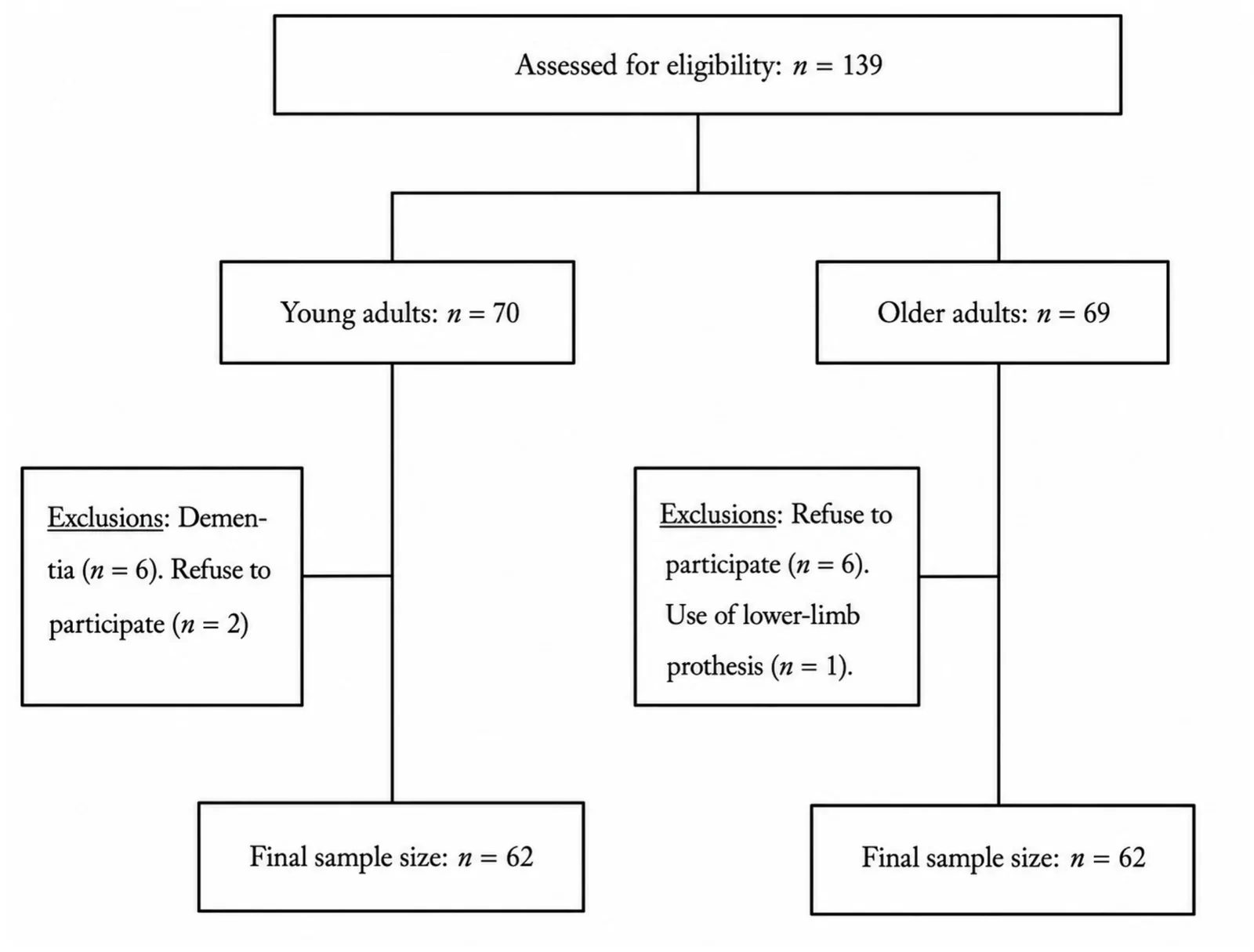
Number of participants in each group.

Individuals were eligible if they were young (18–25 years) or older adults (≥60 years), able to walk independently, had a cell phone, and did not present any musculoskeletal or neurological conditions that could interfere with the gait assessment. The use of participants’ cell phones was adopted to minimize the potential learning effects associated with unfamiliar devices. The exclusion criteria were the presence of neurological or psychiatric disorders, residence in a long-term care facility, and the use of lower limb orthoses or prostheses. Figure 1 shows the number of participants included and excluded during the recruitment process.

The assessments included a sociodemographic questionnaire to characterize the participants according to sex, age, educational level, and body mass index (BMI). Cognitive function was assessed using the Mini-Mental State Examination (MMSE) [35]. The MMSE is a standardized cognitive screening tool that covers multiple cognitive domains, including temporal and spatial orientation, immediate registration and delayed recall of three words, attention and calculation, language, and visuo-constructive abilities. In the MMSE, scores range from 0 to 30, with lower values indicating poorer cognitive performance. After completing the preliminary phases of written informed consent and sociodemographic and cognitive profiles, all participants performed single- and dual-task activities using their cell phones. The order of the activities was randomized to minimize potential learning and sequencing effects.

The timed up and go (TUG) test [36] was used to evaluate motor performance during a dynamic dual-task paradigm involving walking while using a cell phone. The TUG was chosen because of its widespread clinical use in assessing functional mobility and dynamic balance [37]. Unlike isolated gait assessments that focus solely on straight-line walking, the TUG integrates several motor components commonly encountered in daily activities, including sit-to-stand transfer, gait initiation, straight-line walking, turning, deceleration, and stand-to-sit transfer. This approach allowed for a detailed assessment of mobility performance and facilitated the identification of specific gait phases that may be differentially affected by cell phone use.

To examine the influence of cell phone use on mobility performance, the TUG test was administered under one single-task and two dual-task conditions. In the single-task condition, participants were instructed to stand from a chair, walk three meters, turn around, walk back to the chair, and sit down while performing only the mobility task. In the two subsequent dual-task conditions, the participants completed the same TUG protocol while interacting with their own cell phones. The first dual-task condition involved text messaging while performing the TUG test. The participants were instructed to continuously type the following standardized sentence: “Hello, I will be late for my appointment.”

The participants were encouraged to maintain the typing activity for the entire duration of the task without interrupting either the walking or texting performance. This approach was adopted to ensure a continuous cognitive–motor demand while minimizing variability related to message content. The second dual-task condition involved talking on a cell phone while performing the TUG test. To standardize the cognitive demand across participants, a structured conversation was conducted in which participants were asked to recall and describe the meals they had consumed the previous day, including breakfast, lunch, dinner, and snacks. The participants were encouraged to provide detailed descriptions whenever possible, and the examiner used standardized prompts to maintain the conversation throughout the task without introducing additional cognitive challenges or leading the responses.

During all gait assessments, a wearable inertial sensor (BTS Bioengineering, Milan, Italy) was positioned at the L4/L5 lumbar level to record the spatiotemporal and mobility-related parameters. The Baiobit BTS Bioengineering system is a wearable measurement technology used for clinical and research purposes [38]. It includes a lightweight wireless sensor equipped with triaxial accelerometers, gyroscopes, and magnetometers that can continuously track the body movements during functional activities. 

**Figure 2:**
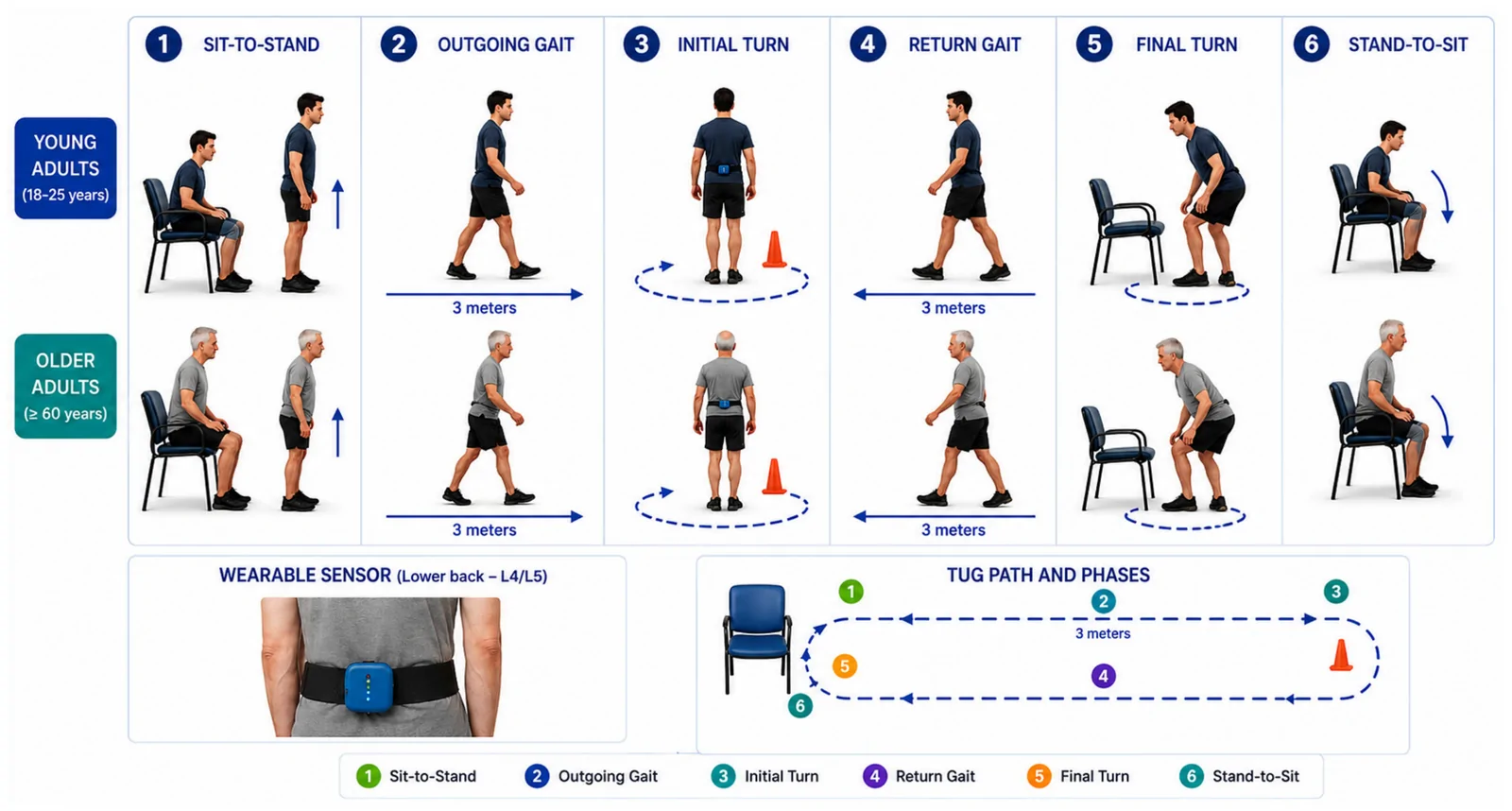
Schematic representation of the TUG test, sensor positioning, and mobility subphases of the TUG test.

The Baiobit BTS Bioengineering system was used because it provides a comprehensive analysis of mobility by automatically detecting and measuring each phase of the TUG test. Figure 2 illustrates the TUG protocol adopted in this study, including the six phases extracted for analysis: sit-to-stand, outgoing gait, initial turn, return gait, final turn, and stand-to-sit position.

### Statistical Analysis

The data were analyzed using R software version 4.4.1 (R Foundation for Statistical Computing, Vienna, Austria). To determine whether the parametric assumptions were satisfied, the distribution of continuous variables was assessed using the Shapiro–Wilk test and Q–Q plots. Since most variables did not satisfy the parametric assumptions, group and pairwise comparisons were performed using nonparametric tests.

Continuous variables are presented as medians and interquartile ranges (interquartile range), and categorical variables are expressed as frequencies and percentages. The Mann–Whitney U test was employed to compare young and older adults, while Friedman’s test was used to compare the experimental conditions (walking without distraction, texting while walking, and talking on the phone while walking). When significant differences emerged between conditions, pairwise comparisons were performed using the Wilcoxon signed-rank test. Effect sizes were estimated using Kendall’s W coefficient. Because age is a well-established determinant of both cognitive function and mobility performance, this variable was included as a covariate in the correlation analyses. Thus, the association between cognitive performance and gait variables was assessed using partial Spearman’s rank correlation coefficients (r_s_) after controlling for age. The correlation coefficients were interpreted as small (0.10 ≤ |rs| < 0.30), moderate (0.30 ≤ |rs| < 0.50), and large (|rs| ≥ 0.50).

Linear regression analysis was performed to identify the predictors of motor performance. Separate regression models were constructed for each dependent variable. A stepwise method was used to select predictors. Prior to model fitting, the assumptions of linear regression were examined, including linearity, homoscedasticity, normality of residuals, and multicollinearity. For each model, standardized regression coefficients (β), 95% confidence intervals (95% CI), coefficient of determination (R^2^), and change in explained variance (ΔR^2^) were reported. The Durbin–Watson test was used to assess the independence of residuals. Variance inflation factors were calculated to assess multicollinearity, with all values indicating an acceptable level of collinearity among predictors. For all analyses, significance was set at 5%.

All the data generated and analyzed during this study are presented in Supplementary Table S1. Due to the substantial volume of information, it was not feasible to include the statistical analyses and derived outputs. Additional details regarding specific analyses can be obtained from the corresponding author upon reasonable request.

## 3. Results

One hundred twenty-four participants were enrolled in this study, comprising 62 young and 62 older adults. The groups were comparable with respect to sample size, sex distribution, and body weight. As expected, older adults were significantly older than young adults. Compared with the older group, young adults were significantly taller, had a lower BMI, and achieved higher MMSE scores (30.0 vs. 28.0 points; *p* < 0.001). Despite this difference, the median MMSE scores of both groups were within the normal range, indicating that all the participants were cognitively preserved. Table 1 summarizes the participants’ demographic, anthropometric, and cognitive characteristics.

Table 2 summarizes mobility performance on the experimental conditions. Older adults exhibited significantly poorer performance than young adults in the total TUG test and in all the subphases of the walking task. Within-group analyses demonstrated that both dual-task conditions significantly impaired mobility compared with the no cell phone condition. In young adults, the total TUG time increased from 9.0 to 9.9 s during talking and to 10.2 s during texting (*p* = 0.001). Older adults required 11.9 s to complete the TUG without a cell phone, increasing to 16.8 s during talking and 17.1 s during texting (*p* = 0.001). Although texting consistently produced longer completion times than talking, post hoc analyses did not reveal significant differences between the two dual-task conditions in either group (*p* > 0.05).

Outgoing gait, initial turn, return gait, and final turn required significantly more time during both talking and texting than during the no cell phone condition in young and older adults (*p* < 0.05 in all comparisons). In contrast, sit-to-stand performance remained unchanged across conditions in both groups (*p* = 0.510 for young adults; *p* = 0.296 for older adults). The stand-to-sit phase was less affected than the walking phases, showing no differences between the no cell phone and talking conditions in young adults, whereas texting significantly increased the completion time. In older adults, both dual-task conditions significantly prolonged the stand-to-sit performance. Across all the analyses, the effect sizes ranged from small to moderate, with the largest effects observed for the total TUG duration, particularly in older adults. The statistical power exceeded 88% and was greater than 99% for most comparisons, supporting the robustness of the findings.

Table 3 presents the correlations between age, MMSE scores, and TUG performance in all three experimental conditions. Age was positively correlated with the total TUG duration in all conditions, with the strongest association observed during the texting condition. Moderate-to-large correlations were identified for the outgoing gait, initial turn, return gait, and final turn, indicating that an increase in age is associated with slower gait and turning performance. These associations were stronger under dual-task conditions, particularly during texting, suggesting that greater cognitive demands accentuate the influence of aging on mobility. In all the TUG subphases, the turning components exhibited the strongest age-related correlations, highlighting turning as one of the mobility tasks most affected by aging.

MMSE scores were negatively correlated with the TUG outcomes, indicating that better global cognitive performance was associated with shorter execution times. Significant correlations were observed for the total TUG duration in all the conditions, with the strongest association identified during the talking condition. Moderate negative correlations were observed for the outgoing gait, initial turn, return gait, final turn, and stand-to-sit phase, indicating that better cognitive function was consistently associated with faster mobility performance. The sit-to-stand phase was the only TUG component that was not significantly associated with either age or MMSE in any of the experimental conditions, suggesting that this relatively simple movement may be less influenced by age- or cognition-related factors than the more dynamic gait and turning phases.

To investigate the relative contributions of age and cognition to mobility performance, multiple linear regression analyses were performed using age and MMSE scores as potential predictors of TUG outcomes. Age emerged as the primary predictor of the total TUG duration and most gait and turning-related components in the three experimental conditions. For total TUG time, age explained between 27.3% and 42.4% of the variance, with the strongest predictive effect observed during the texting condition. These findings indicate that age-related factors influence mobility performance, particularly when walking is combined with cognitively demanding cell phone-related tasks. In the no cell phone condition, the MMSE contributed to the prediction of total TUG duration, increasing the explained variance in mobility by 2.4%. Age was the main predictor of outgoing gait, return gait, initial turn, and final turn durations, accounting for 20–35% of the variance in mobility outcomes. The predictive influence of age was particularly evident during the turning phases, which demonstrated some of the highest R^2^ values across analyses. The initial turn performed without cell phone use was the only turning phase in which the MMSE contributed independently to the model, explaining 2.8% of the variance of mobility. Table 4 presents the results of the regression analyses examining the influence of age and MMSE scores on the TUG performance.

Unlike age, cognitive performance was more strongly associated with transitional movements. The MMSE was the only significant predictor of sit-to-stand duration in the no-cell-phone condition and of stand-to-sit duration in the no-cell-phone and talking conditions. For stand-to-sit performance during the talking condition, both MMSE and age contributed significantly to the final model, indicating that this phase may be influenced by both cognitive and age-related factors. Nevertheless, the contribution of cognitive function was smaller than that of age, suggesting that global cognitive status plays an important but secondary role in explaining dual-task mobility performance in cognitively preserved individuals. Overall, the findings presented in Table 4 indicate that although cognition contributes to specific mobility components, particularly transitional movements, the effects of aging exert a broader and more consistent influence on functional mobility under both single- and dual-task conditions.

## 4. Discussion

This study investigated the effects of cell phone use on mobility in young and older adults by examining both overall TUG performance and its individual components. Three main findings were obtained in this study. First, older adults demonstrated poorer mobility than young adults across all experimental conditions. Second, both phone conversations and text messaging significantly impaired TUG performance, increasing the time required to complete the task. Third, age was the strongest determinant of mobility performance in all TUG phases, whereas global cognitive function showed a smaller but significant contribution to the selected mobility components. Together, these findings suggest that cell phone dual-tasking substantially interferes with motor control and that aging exerts a stronger influence on mobility performance than cognition in healthy individuals.

The detrimental effects of cell phone use on mobility are consistent with the dual-task interference framework [39,40,41]. Walking is often considered an automated motor behavior. However, accumulating evidence indicates that successful locomotion requires continuous integration of sensory information, attentional resources, executive control, and anticipatory motor planning. When individuals simultaneously engage in cell phone-related activities, their attentional resources are divided between locomotor control and cognitive processing. According to capacity-sharing models, the CNS has limited attentional reserves, and competition between concurrent tasks results in reduced performance in one or both activities [42]. The significant increases observed in the total TUG duration during both talking and texting conditions support this interpretation and indicate that cell phone use imposes sufficient cognitive demands to disrupt mobility performance.

Interestingly, although texting produced numerically greater impairments than phone conversations, post hoc analyses did not reveal any significant differences between the two dual-task conditions. This finding suggests that both cell phone activities generate meaningful cognitive–motor interference despite their distinct cognitive requirements. Texting typically involves visual attention, language processing, working memory, and fine motor control of the fingers, whereas phone conversations primarily require auditory processing, language production, and divided attention [43]. Although texting is generally considered a more cognitively demanding task, the present results indicate that even conversational cell phone use is sufficient to compromise mobility, particularly in older adults. These findings reinforce concerns regarding the widespread use of cell phones during walking and highlight the potential risks of its use while being distracted by a secondary task.

Analysis of the TUG subphases provides important biomechanical insights into how cell phone use during dual-tasking affects locomotor control. The outgoing gait phase was significantly prolonged during both dual-task conditions in young and older adults. This phase reflects forward progression immediately after gait initiation and requires continuous regulation of step length, cadence, and dynamic balance. Increased duration of cell phone use suggests a reduction in gait speed, likely representing a compensatory strategy to preserve stability when attention is diverted toward a secondary task. Such adaptations are commonly observed during cognitively demanding walking tasks and may reflect an attempt to reduce instability and minimize the risk of falling [44].

Turning phases were among the most affected components of the TUG test. Both the initial and final turns demonstrated significant increases in execution time across conditions, with particularly pronounced impairments in older adults. Turning is considered one of the most complex walking tasks because it requires anticipatory postural adjustments, precise control of the center of mass, rapid reorientation of body segments, and continuous sensorimotor integration [45]. Unlike straight-line walking, turning requires coordinated movements of the head, trunk, pelvis, and lower limbs while simultaneously maintaining a dynamic balance. The larger effects observed during the turning phases are consistent with the greater attentional and motor-control demands associated with these tasks, which may make them more susceptible to dual-task interference. This finding is relevant because turning deficits are strongly associated with falls and mobility limitations in older adults [46].

The return gait phase exhibited substantial deterioration during dual-task performance. This phase combines the biomechanical demands of straight-line walking with the residual effects of the preceding turn, requiring continuous adaptation of the gait parameters. The prolonged duration of cell phone use may reflect increased caution, reduced gait automaticity, and a greater reliance on conscious motor control. Previous studies have shown that walking in diverse situations increases activation in the frontal cortical regions involved in executive processing, supporting the notion that locomotion becomes less automatic when cognitive resources are shared between tasks [46,47,48]. In contrast, the sit-to-stand phase was relatively unaffected by cell phone use and showed no significant correlation with age or cognitive function. Sit-to-stand performance depends mainly on lower limb muscle strength, momentum generation, and biomechanical efficiency rather than on continuous environmental monitoring. Because the movement is brief and highly practiced, it may require fewer attentional resources than gait and turning movements. Consequently, the dual-task costs associated with cell phone use appear to have a limited influence on this transitional movement.

The stand-to-sit phase exhibited a different pattern. Significant impairments were observed, particularly in older adults, and both age and cognition contributed to performance in certain conditions. Descending into a seated position requires precise modulation of eccentric muscle activity, anticipatory control of body momentum, and an accurate estimation of chair location. These processes depend on sensory integration and executive control, making the task more susceptible to cognitive interference than the sit-to-stand transition [49].

The correlation analyses emphasized the dominant role of aging in mobility. Age demonstrated moderate-to-strong associations with total TUG duration and with most gait and turning components across all experimental conditions. Notably, the strongest correlations were observed during texting, suggesting that age-related changes become increasingly relevant as task complexity increases. Several physiological mechanisms may explain these findings. Aging is associated with declines in muscle strength, sensory function, processing speed, reaction time, and postural control. Structural and functional changes in the cortical and subcortical networks also reduce the efficiency of motor planning and sensorimotor integration. Consequently, older adults possess fewer reserve resources to manage the additional demands imposed by cell phone-related dual-tasking [50].

Although weaker than age-related effects, this study showed that cognitive performance is associated with mobility. Higher MMSE scores were consistently related to shorter TUG durations, particularly for the turning phases and total test performance. These findings support previous evidence linking cognition and mobility through shared neural substrates [25,51]. Executive functions, attention allocation, working memory, and information processing speed contribute substantially to successful navigation during complex walking tasks. Even in a cognitively preserved sample, subtle differences in global cognitive performance appear to influence mobility efficiency when attention demands increase.

Regression analyses provided further insights into the contributions of aging and cognition to mobility control. Age emerged as the primary predictor of total TUG duration and most gait- and turning-related components, explaining up to 42.4% of the variance in the motor performance. Particularly noteworthy was the finding that age accounted for greater variability during cell phone-related dual-tasking than during normal walking. This suggests that age-related physiological changes reduce the capacity to cope with competing cognitive and motor demands, thereby amplifying the dual-task cost. From a neuroscience perspective, these findings align with theories proposing reduced neural efficiency, diminished processing capacity, and decreased network flexibility with increasing age [52].

Different than age, cognitive performance contributed to only a limited number of outcomes, such as sit-to-stand and stand-to-sit movements. Furthermore, the additional variance explained by the MMSE scores was relatively small. These findings indicate that, in cognitively preserved individuals, chronological age has a stronger influence on mobility performance than global cognitive status. Nevertheless, the contribution of cognition to the selected TUG phases suggests that executive and attentional processes are important determinants of mobility.

From a Brain Sciences perspective, this study reinforces the concept that mobility represents a complex neurocognitive behavior rather than a purely motor task. The influence of age on gait and turning performance is consistent with previous evidence indicating that locomotor control depends on the integration of cognitive and motor-control systems [50,51,52]. Likewise, the modest but significant contribution of cognition is consistent with the role of executive functioning in mobility during complex daily activities. Although the findings support the existence of cognitive–motor interference during cell phone use, they do not allow direct inferences regarding the underlying neural mechanisms because only behavioral outcomes were assessed. Future studies incorporating executive-function testing together with neurophysiological and neuroimaging techniques are needed to clarify these mechanisms.

A factor that may have contributed to the observed age-group differences is the participants’ familiarity and proficiency with cell phone use. Younger adults generally use cell phones more frequently and may therefore perform texting and phone conversation tasks more automatically, requiring fewer attentional resources than older adults. Consequently, part of the greater dual-task cost observed in older participants may reflect differences in cell phone experience rather than aging-related changes alone. Although cell phone proficiency was not quantified in this study, this potential confounding factor should be considered when interpreting the magnitude of the age-related effects. Future studies should assess smartphone use habits and digital literacy to better distinguish the influence of technological familiarity from the physiological effects of aging.

### Limitations

This study has several limitations that should be acknowledged. First, its cross-sectional design precludes causal inferences regarding the relationships between age, cognitive function, cell phone use, and mobility performance. Second, cognitive status was assessed using the MMSE, a global cognitive screening tool that may not fully capture specific domains, such as executive function, attention, processing speed, and working memory. Third, although the texting and phone conversation tasks were standardized, the participants’ frequency of cell phone use and texting habits were not recorded. Consequently, individual differences in familiarity with cell phone use and proficiency in texting may have influenced performance. Finally, this study relied on clinical measures without incorporating neurophysiological or neuroimaging biomarkers. Although this approach is appropriate for addressing the study objectives and reflects assessments commonly used in clinical practice, it does not allow for the direct investigation of the neural mechanisms underlying age-related dual-task interference. Future studies combining behavioral assessments with techniques such as electroencephalography, electromyography, and functional neuroimaging may provide a more comprehensive understanding of the neural and physiological processes associated with mobility during cell phone-related dual-tasking.

## 5. Conclusions

Cell phone use while walking negatively affects mobility performance, with older adults exhibiting greater impairment than young adults. Both texting and phone conversations increased cognitive–motor interference and worsened gait-related outcomes. These findings are consistent with increased demands on attentional and motor-control systems involved in attentional allocation, executive functioning, and motor control, particularly in older adults.

Age emerged as the primary determinant of mobility performance, explaining a substantial proportion of the variability in total TUG duration and its subcomponents, whereas global cognitive function provided a smaller but independent contribution to the selected transitional movements. These results highlight the importance of considering both aging and cognitive factors when developing strategies to promote safe mobility and reduce the risks associated with cell phone use while walking.

## Figures and Tables

**Table 1:** Anthropometric and clinical characteristics of the participants.

Variables	Young Adults	Old Adults	*p*
Sample size, *n*	62	62	0.999
Sex (male:female), *n*	13:49	11:51	0.649
Age, years	25.0 (7.0)	67.0 (8.0)	0.001
Weight, kg	70.5 (19.8)	72.0 (21.5)	0.586
Height, m	1.68 (0.1)	1.60 (0.1)	0.001
BMI, kg/m^2^	25.0 (4.5)	27.3 (6.70)	0.015
MMSE, pts	30.0 (1.0)	28.0 (2.0)	0.001

BMI: Body mass index; MMSE: Mini-Mental State Examination. Data are presented in median (interquartile range). The Mann–Whitney U test was used for continuous variables, and the chi-square for categorical variables.

**Table 2:** Timed up and go test performance and subcomponents during no cell phone, phone conversation, and text messaging conditions in young and older adults.

Variables	Groups	Tasks			*P*	Effect Size	Power (%)
		No Cell Phone	Talking on Phone	Texting Messages			
TUG test, s	Young adults	9.0 (2.6) ^a^	9.9 (3.0) ^b^	10.2 (3.0) ^c^	0.001	0.266	99.9
	Older adults	11.9 (4.9) ^a^	16.8 (6.5) ^b^	17.1 (6.3) ^c^	0.001	0.572	99.9
	*p*	0.001	0.001	0.001			
Sit-to-stand, s	Young adults	1.6 (0.5) ^a^	1.8 (0.5) ^a^	1.6 (0.5) ^a^	0.510	NS	NS
	Older adults	1.5 (0.8) ^a^	1.7 (0.7) ^a^	1.6 (0.9) ^a^	0.296	NS	NS
	*p*	0.250	0.863	0.321			
Outgoing gait, s	Young adults	1.8 (0.8) ^a^	2.1 (3.2) ^b^	2.3 (1.0) ^b^	0.001	0.174	99.7
	Older adults	2.7 (1.3) ^a^	3.7 (3.2) ^b^	4.0 (2.5) ^b^	0.001	0.291	99.9
	*p*	0.001	0.001	0.001			
Initial turn, s	Young adults	1.2 (0.3) ^a^	1.3 (0.3) ^b^	1.4 (0.6) ^b^	0.013	0.091	88.5
	Older adults	1.9 (0.7) ^a^	2.2 (1.0) ^b^	2.3 (1.1) ^b^	0.001	0.154	99.1
	*p*	0.001	0.001	0.001			
Return gait, s	Young adults	1.9 (1.1) ^a^	2.5 (1.2) ^b^	2.4 (1.3) ^b^	0.001	0.324	99.9
	Older adults	2.7 (1.9) ^a^	4.7 (3.5) ^b^	4.4 (1.4) ^b^	0.001	0.345	99.9
	*p*	0.001	0.001	0.001			
Final turn, s	Young adults	0.9 (0.2) ^a^	1.1 (0.3) ^b^	1.0 (0.3) ^b^	0.001	0.123	94.8
	Older adults	1.5 (0.6) ^a^	2.0 (0.8) ^b^	1.8 (0.8) ^b^	0.001	0.184	99.8
	*p*	0.001	0.001	0.001			
Stand-to-sit position, s	Young adults	1.0 (0.5) ^a^	1.0 (0.8) ^a^	1.3 (0.6) ^b^	0.004	0.161	99.4
	Older adults	1.2 (0.7) ^a^	1.5 (1.1) ^b^	1.8 (1.5) ^b^	0.001	0.205	99.9
	*p*	0.213	0.001	0.001			

TUG: Timed up and go. NS: comparison not statistically significant (*p* ≥ 0.05). Different superscript letters (a–c) within the same row denote statistically significant pairwise differences between task conditions (*p* < 0.05). Identical letters indicate no significant difference.

**Table 3:** Correlations of age and MMSE scores with TUG across single-task and cell phone dual-task conditions.

Walking Phase	Variables	Tasks		
		No Cell Phone	Talking on Phone	Texting Messages
TUG total test	Age	0.522	0.630	0.651
	Cognition	−0.461	−0.492	−0.453
Sit-to-stand	Age	NS	NS	NS
	Cognition	NS	NS	NS
Outgoing gait	Age	0.449	0.532	0.486
	Cognition	−0.292	−0.413	−0.297
Initial turn	Age	0.569	0.590	0.576
	Cognition	−0.502	−0.405	−0.483
Return gait	Age	0.445	0.530	0.505
	Cognition	−0.352	−0.400	−0.380
Final turn	Age	0.556	0.593	0.467
	Cognition	−0.347	−0.432	−0.278
Stand-to-sit position	Age	0.207	0.402	0.474
	Cognition	−0.209	−0.408	−0.394

TUG: Timed up and go. NS = non-significant (*p* ≥ 0.05). All reported correlation coefficients are statistically significant (*p* < 0.05) unless otherwise indicated.

**Table 4:** Regression models examining the contributions of age and cognition to TUG performance under single-task and dual-task conditions.

Variables	Condition	Models	Predictors	β	*p*	95% CI	R^2^	VIF	Durbin–Watson
TUG total test	No cell Phone	1	Age	0.386	0.001	0.029 to 0.093	0.273	1.779	1.753
		2	Age + cognition	−0.206	0.005	−0.927 to −0.10	0.024	1.779	
	Talking on Phone	1	Age	0.630	0.001	0.133 to 0.209	0.397	1.000	1.567
	Texting Messages	1	Age	0.651	0.001	0.170 to 0.261	0.424	1.000	1.572
Sit-to-stand	No cell Phone	1	Cognition	−0.220	0.014	−0.200 to −0.023	0.049	1.000	1.731
Outgoing gait	No cell Phone	1	Age	0.449	0.001	0.016 to 0.033	0.202	1.000	2.108
	Talking on Phone	1	Age	0.532	0.001	0.035 to 0.062	0.283	1.000	1.643
	Texting Messages	1	Age	0.486	0.001	0.046 to 0.090	0.236	1.000	1.729
Initial turn	No cell Phone	1	Age	0.422	0.001	0.006 to 0.017	0.324	1.779	2.104
		2	Age + cognition	−0.223	0.024	−0.163 to −0.012	0.028	1.779	
	Talking on Phone	1	Age	0.590	0.001	0.018 to 0.030	0.348	1.000	1.932
	Texting Messages	1	Age	0.576	0.001	0.024 to 0.040	0.332	1.000	1.625
Return gait	No cell Phone	1	Age	0.445	0.001	0.016 to 0.034	0.198	1.000	1.485
	Talking on Phone	1	Age	0.530	0.001	0.046 to 0.084	0.281	1.000	1.528
	Texting Messages	1	Age	0.505	0.001	0.056 to 0.105	0.255	1.000	1.629
Final turn	No cell Phone	1	Age	0.556	0.001	0.009 to 0.015	0.309	1.000	1.581
	Talking on Phone	1	Age	0.593	0.001	0.015 to 0.025	0.352	1.000	1.563
	Texting Messages	1	Age	0.468	0.001	0.014 to 0.028	0.218	1.000	0.758
Stand-to-sit position	No cell Phone	1	Cognition	−0.209	0.020	−0.136 to −0.012	0.044	1.000	2.090
	Talking on Phone	1	Cognition	−0.253	0.021	−0.287 to −0.023	0.166	1.779	1.901
		2	Cognition + Age	0.234	0.033	0.001 to 0.019	0.031	1.779	
	Texting Messages	1	Age	0.474	0.001	0.014 to 0.028	0.225	1.000	1.894

Only significant predictors are presented. No significant models were observed for sit-to-stand performance during the talking and texting conditions. β: standardized regression coefficient; CI: confidence interval; R^2^: coefficient of determination; VIF: variance inflation factor.

## Data Availability

All the raw data generated and analyzed during this study are presented in Supplementary Table S1. Additional details regarding specific analyses can be obtained from the corresponding author upon reasonable request.

## Supplementary Materials

The following supporting information can be downloaded at: https://www.mdpi.com/article/10.3390/brainsci16080807/s1, Table S1: Raw data generated and analyzed during this study.
