# Supplementary material for: Understanding the Cognitive Load of Cell Phone Use While Walking: Distinct Effects of Texting and Phone Conversation
Source: Brain Sci. 2026 Jul 30;16(8):807. doi: 10.3390/brainsci16080807 (PMC13511484; doi:10.3390/brainsci16080807)
Supplement: Supplementary file 1 [file brainsci-16-00807-s001.zip › brainsci-4422673-supplementary.pdf]

Supplementary Table S1. Raw data generated and analyzed during this study

Group	Sex	Age	Weight	Height	MMSE	TUG simple (s)	TUG texting (s)	TUG talking (s)
2	2	50	64	156	28	10,91	9,85	11,56
2	1	25	77	185	30	8,71	8,62	7,94
2	1	20	67	170	30	11,46	14,56	12,67
2	1	18	70	176	29	7,7	7,88	9
2	2	32	58	157	29	6,79	7,21	8,1
2	1	22	94	185	30	7,66	8,48	6,2
2	2	48	93	160	27	21,11	14,82	16,21
2	2	22	63	162	30	9,24	8,04	9,03
2	2	33	69	167	30	5,51	10,48	11,49
2	1	24	85	179	30	9,68	12,25	12,03
2	2	29	85	178	29	11,15	12,25	15,2
2	2	26	44	160	29	12,74	15,2	15,25
2	2	25	69	170	29	10,74	13,73	13,43
2	2	20	60	163	30	10,95	12,64	13,12
2	1	22	92	178	30	7,59	9,12	7,77
2	2	26	47	154	30	10,43	11,21	12,7
2	1	27	75	181	30	7,74	10,63	11,53
2	2	25	67	167	30	8,81	10,4	8,97
2	2	33	79	173	30	9,15	10,38	9,29
2	2	34	70	170	29	10,06	10,68	9,82
2	2	37	71	163	30	8,24	9,04	8,28
2	1	27	78	172	30	10,47	13,32	9,86
2	2	21	55	163	30	9,37	11,5	13,11

2	2	22	94	167	30	10,39	11,61	12,09
2	2	26	89	166	29	10,88	13,91	14,83
2	2	35	70	157	30	8,47	10,3	8,87
2	1	35	120	181	30	7,24	7,69	9,04
2	1	22	86	180	29	8,01	8,67	8,99
2	2	26	78	164	30	10,25	10,61	9,57
2	2	18	80	163	30	9,02	15,9	10,77
2	1	35	88	176	30	7,05	8,7	10,28
2	2	26	101	173	29	9,4	9,94	11,46
2	1	37	82	168	30	8,31	8,83	8,24
2	2	48	67	169	30	9,1	10,25	10,64
2	2	25	57	159	29	10,49	10,71	11,92
2	1	18	72	168	30	8,16	11,8	10,33
2	2	25	104	165	29	10,99	12,71	14,66
2	2	22	77	169	29	10,69	13,79	13,03
2	2	21	54	153	30	9,02	11,8	11,5
2	2	23	82	170	30	9,87	11,59	9,65
2	2	19	70	167	30	11,46	14,56	12,67
2	2	21	85	178	30	7,7	7,88	9
2	2	24	65	167	30	7,66	8,04	9,03
2	2	27	70	171	30	9,24	8,04	9,03
2	2	22	60	162	30	6,79	7,21	8,1
2	2	28	83	169	29	10,91	9,85	11,56
2	2	30	58	157	30	8,71	8,62	7,94
2	2	25	75	168	30	7,5	9,8	11,5
2	2	29	78	169	29	8,2	10,1	12
2	2	20	64	162	30	7,8	10,3	12,5
2	2	20	70	169	30	7,7	7,88	9
2	2	21	60	165	30	10,95	11,64	12,12

2	2	27	72	173	30	7,7	7,88	9
2	2	30	68	165	30	8,81	10,4	8,97
2	2	21	87	181	30	8,01	8,67	8,99
2	2	28	78	172	30	8,31	8,83	8,24
2	2	21	54	156	29	8,01	8,67	8,99
2	2	20	59	169	30	9,24	8,04	9,03
2	2	19	62	167	30	7,89	8,43	9,78
2	2	27	87	175	30	9,4	9,94	11,46
2	2	28	69	168	29	10,06	10,68	9,82
2	2	30	59	167	30	9,89	10,05	9,96
1	2	61	86	170	30	8,98	19,69	16,98
1	2	60	57	165	30	7,22	16,59	10,39
1	2	63	76	159	28	10,13	17,36	16,15
1	2	61	61	169	27	11,09	20,46	18,08
1	2	71	57	164	28	13,98	16,65	16,31
1	2	61	93	160	27	12,11	14,82	16,21
1	2	60	64	156	29	10,91	9,85	11,56
1	2	65	84	158	30	15,54	20,29	16,88
1	1	66	86	173	30	10,45	15,47	13,25
1	2	81	70	167	29	11,59	16,44	16,05
1	2	63	55	153	29	9,72	11,96	10,81
1	2	64	97	150	30	14,23	16,58	14,1
1	2	68	68	153	28	17,55	43	23,13
1	2	78	57	154	27	16,9	27,32	25,02
1	2	65	80	145	27	14,6	21,21	23,91
1	2	77	68	160	27	18,31	24,76	27,22
1	2	69	54	155	29	18,67	30,71	27,48
1	2	72	84	165	27	10,93	24,56	14,54
1	2	65	72	158	27	13,81	22,14	24,11

1	2	75	77	161	29	29,42	50,95	50,59
1	2	71	81	156	28	13,59	18,28	15,97
1	2	62	70	155	27	15,41	19,79	20,68
1	2	72	75	160	27	16,94	32,63	20,99
1	2	71	76	151	27	16,4	27,84	25,12
1	2	68	80	160	28	13,43	21,12	16,37
1	2	61	78	145	30	9,65	12,35	10,19
1	2	70	57	150	28	18,13	25,95	25,59
1	2	70	60	162	28	13,98	28,3	18,33
1	2	63	83	160	28	10,84	13,75	16,82
1	2	66	98	153	27	11,45	14,17	16,94
1	2	68	53	155	28	9,74	14,98	16,71
1	2	63	76	159	27	15,49	25,79	25,56
1	2	81	70	167	27	18,48	36,76	27,98
1	2	73	56	150	21	12,83	16,02	17,6
1	2	66	75	160	28	16,94	32,63	20,99
1	2	80	65	156	27	8,98	19,69	16,98
1	1	74	103	175	29	8,85	13,59	12,86
1	2	68	78	163	29	13,98	16,65	14,88
1	2	87	43	145	24	11,98	20,98	17,98
1	1	61	72	172	27	13,98	20,98	22,98
1	2	60	80	164	30	11,98	14,98	17,98
1	2	64	70	162	27	9,98	17,98	17,98
1	2	60	52	157	29	9,6	12,98	11,52
1	1	61	83	182	28	9,43	19,02	17
1	1	79	80	170	28	11,75	17,58	17,98
1	1	69	100	170	27	7,92	12,71	9,6
1	2	66	58	158	29	7,98	10,74	8,98
1	1	64	75	172	30	9,75	14,86	16,98

1	1	68	58	165	26	11,98	18,83	14,98
1	2	67	61	156	27	9,98	15,98	13,98
1	2	68	70	165	27	9,6	14,77	14,98
1	2	68	85	153	26	16,98	19,85	20,86
1	1	69	61	168	28	12,98	16,98	15,42
1	2	63	52	160	27	8,98	12,58	9,87
1	2	66	74	156	29	9,87	11,98	10,98
1	2	70	57	150	24	14,98	17,98	15,98
1	2	71	67	150	29	7,98	10,97	12,68
1	1	64	83	175	27	14,5	15,86	20,32
1	2	71	61	157	27	12,82	16,48	14,31
1	2	62	60	164	27	10,97	10,54	13,77
1	2	64	93	164	27	12,98	15,98	18,98
1	1	67	110	183	28	8,98	13,76	15,98

**Sit-to-stand simple task (s)**	**Outgoing gait simple task (s)**	**Initial turn simple task (s)**	**Return gait simple task (s)**	**Final turn simple task (s)**	**Stand-to-sit simple task (s)**	**Sit-to-stand texting (s)**	**Outgoing gait simple texting (s)**	**Initial turn texting (s)**	**Final turn texting (s)**
1,99	1,93	1,83	2,99	0,86	1,31	1,33	1,88	1,42	3,23
1,46	1,09	0,87	1,87	2,01	1,26	1,99	1,75	1,12	2,1
1,14	2,93	1,27	2,85	1,93	1,34	0,98	5,42	1,49	4,55
1,38	1,92	0,83	1,97	0,87	0,73	1,42	2,41	0,94	2,18
1,31	1,5	1,35	1,56	0,66	0,41	2,31	1,54	0,69	1,82
1,89	1,4	1,22	1,39	1,04	0,72	1,04	1,51	0,4	2,99
0,86	2,5	2,32	3,87	1,54	1,02	2,48	3,55	2,01	3,81
1,64	1,63	1,24	3,08	0,86	0,79	1,59	1,73	1,05	2,23
0,51	0,87	0,79	1,42	0,98	0,94	1,53	1,88	1,25	3,46
0,67	2,98	1,24	1,74	1,37	1,68	1,68	3,17	1,53	3,29

1,82	2,55	1,14	2,82	1,1	1,72	1,56	4,06	1,84	3,54
1,43	2,84	2,13	3,56	1,24	1,54	0,56	4,57	2,79	5,63
2,28	2,17	1,08	3,13	1,04	1,04	1,62	2,3	1,98	5,33
1,08	3,17	1,29	2,87	0,99	1,55	1,7	3,08	2,13	3,81
1,87	0,99	1,15	1,68	0,97	0,93	1,96	1,4	1,11	2,06
1,65	2,78	1,45	2,65	0,78	1,12	1,66	3,18	1,99	2,43
2,75	0,53	0,94	2	0,76	0,76	2,29	1,83	1,26	2,99
1,25	2,33	1,35	1,85	1,08	0,95	2,12	1,73	1,92	2,43
1,86	1,13	1,21	2,77	1,54	0,64	1,49	2,49	1,43	2,21
2,12	1,84	1,84	1,99	1,02	1,25	1,63	2,73	1,25	3,05
1,95	1,39	1,35	1,61	0,77	1,17	0,74	2,93	1,24	1,89
2,25	1,72	1,49	2,02	1,18	1,81	1,76	2,9	2,15	3,97
1,47	2,06	1,08	2,12	1,16	1,48	2	2,47	1,75	2,77
1,6	2,32	1,08	2,5	1,42	1,47	1,68	2,37	1,68	3,57
1,32	2,6	1,48	3,09	1,06	1,33	1,88	3,79	1,92	3,78
1,19	1,95	1,37	1,63	1,14	1,19	1,23	2,64	1,73	2,43
1,85	1,05	1,08	1,19	0,85	1,22	1,56	2,58	0,54	1,85
1,8	1,61	1,32	1,5	0,85	0,93	2,01	1,4	1,25	1,6
1,72	2,34	1,55	1,79	0,98	1,87	1,51	2,73	1,56	1,87
2,3	1,73	1,06	2,37	1,11	0,45	2,01	5,13	1,74	4,29
0,92	1,06	1,01	1,22	0,95	1,35	1,48	1,93	1,25	2,16
1,53	1,92	1,36	1,88	0,83	1,88	1,12	2,75	1,43	2,16
1,46	1,71	1,27	2,02	0,92	0,93	1,99	1,47	1,37	2,1
1,76	1,79	1,49	1,57	1,07	1,42	1,91	2,09	1,69	1,65
1,71	1,87	1,34	2,17	1,87	1,53	1,49	1,95	1,45	3,52
0,73	2,63	1,28	1,64	0,95	0,93	1,34	2,72	1,67	3,4
1,21	2,72	1,35	2,88	1,18	1,65	1,72	2,68	1,53	3,43
1,68	2,53	1,15	2,88	1,07	1,38	1,96	3,67	1,91	4,1
1,52	1,77	1,45	1,94	1,03	1,31	1,45	2,37	1,53	4,52

1,71	1,97	1,37	1,94	1,18	1,7	1,5	2,95	1,78	2,57
2,14	2,93	1,27	2,85	0,93	1,34	0,98	5,42	1,49	4,55
1,38	1,92	0,83	1,97	0,87	0,73	3,42	0,41	0,94	2,18
1,89	1,4	1,22	1,39	1,04	0,72	1,59	1,73	1,05	2,23
1,64	1,63	1,24	3,08	0,86	0,79	1,59	1,73	1,05	2,23
1,31	1,5	1,35	1,56	0,66	0,41	3,31	0,54	0,69	1,82
1,99	1,93	1,83	2,99	0,86	1,31	1,33	1,88	1,42	3,23
2,46	1,09	0,87	2,01	1,26	1,02	1,99	1,75	1,12	2,1
1,2	1,5	1	2	0,7	0,8	1,5	2	1,3	2,5
1,3	1,6	1,1	2,2	0,8	0,9	1,6	2,1	1,4	2,7
1,25	1,55	1,05	2,05	0,75	0,85	1,55	2,05	1,35	2,55
1,38	1,92	0,83	1,97	0,87	0,73	1,42	2,41	0,94	2,18
1,08	3,17	1,29	2,87	0,99	1,55	1,7	2,08	2,13	3,81
1,38	1,92	0,83	1,97	0,87	0,73	1,42	2,41	0,94	2,18
1,25	2,33	1,35	1,85	1,08	0,95	2,12	1,73	1,92	2,43
1,8	1,61	1,32	1,5	0,85	0,93	2,01	1,4	1,25	1,6
1,46	1,71	1,27	2,02	0,92	0,93	1,99	1,47	1,37	2,1
1,8	1,61	1,32	1,5	0,85	0,93	2,01	1,4	1,25	1,6
1,64	1,63	1,24	3,08	0,86	0,79	1,59	1,73	1,05	2,23
1,89	1,4	1,22	1,39	1,04	0,95	1,52	1,76	1,09	2,38
1,53	1,92	1,36	1,88	0,83	1,88	1,12	2,75	1,43	2,16
2,12	1,84	1,84	1,99	1,02	1,25	1,63	2,73	1,25	3,05
1,76	1,23	1,32	2,65	1,55	1,38	1,35	2,49	1,47	2,3
0,67	2,7	1,6	0,93	1,11	1,97	1,9	5,51	1,93	1,04
0,58	2,1	1,1	0,87	0,98	1,59	1,74	4,98	1,91	0,99
0,78	2,9	1,8	1,16	1,39	2,1	1,94	5,1	2,4	1,9
0,8	2,5	1,8	1,7	1,97	2,32	2,1	5,7	3,1	2,4
1,57	3,02	2,42	1,22	2,87	2,88	1,03	3,98	2,58	1,46
0,86	2,5	3,87	1,02	2,32	1,54	2,48	3,55	3,81	1,43

1,99	1,93	2,99	1,31	1,83	0,86	1,33	1,88	3,23	0,92
3,6	2,89	4,04	1,57	1,95	1,49	1,53	5,76	6,87	2,93
1,61	2,82	2,67	0,24	1,98	1,13	0,77	4,07	5,32	1,44
0,7	2,8	1,5	1,92	1,94	2,73	2,74	3,78	4,06	1,89
1,11	2,46	2,47	0,91	1,51	1,26	1,46	3,57	3,38	0,85
1,54	3,72	1,79	1,44	1,95	1,79	2,83	4,55	3,43	2,59
0,49	2,23	5,18	0,64	0,2	1,99	2,13	18,91	14,47	2,39
2,29	2,87	4,78	2,17	2,96	1,83	2,43	8,45	7,54	3,35
1,53	3,68	4,38	1,84	1,71	1,46	1,12	6,76	7,6	1,91
1,79	5,14	5,09	2,56	1,98	1,75	2,2	7,41	8,58	2,48
2,26	4,7	6,34	1,59	1,98	1,8	1,99	12,25	11,2	1,72
1,38	3,43	2,67	0,85	1,39	1,21	0,57	3,53	20,72	1,1
5,69	1,16	3,82	1,59	1,17	1,38	2,13	5,97	6,79	3,15
2,38	8,75	8,86	2,84	3,95	2,64	4,13	15,52	21,67	3,42
1,76	3,55	3,22	1,79	1,31	1,96	2,28	4,71	5,46	2,52
2,06	2,6	4,94	1,74	2,53	1,54	2,56	4,56	5,68	2,09
1,76	4,83	4,54	3,03	1,34	1,44	1,48	9,93	12,73	5,13
1,15	6,52	4,81	1,21	1,79	0,92	2,33	9,07	8,93	2,23
3,47	2,51	4,48	1,16	1,33	0,48	1,33	1,6	9,46	1,43
1,59	1,93	2,49	1,08	1,42	1,14	1,36	3,2	3,35	1,46
2,16	5,6	5,03	1,99	1,61	1,74	3,59	7,43	7,8	2,83
1,75	2,49	5,15	1,37	1,98	1,24	2,04	4,52	12,9	1,95
1,5	2,65	2,35	1,57	1,44	1,33	1,96	3,66	3,35	1,58
1,15	2,82	3,06	1,57	1,49	1,36	1,2	3,23	4,53	0,9
1,85	1,38	2,57	1	1,68	1,25	1,45	3,83	2,62	1,38
4,44	1,72	5,33	0,87	2,45	0,68	2,62	5,35	7,24	5,54
1,45	6,52	5,16	2,3	2,04	1,01	2,03	21,06	5,48	0,2
4,81	1,45	2,75	0,65	1,62	1,55	2,19	5,16	2,77	1,75
1,76	4,83	4,54	3,03	1,34	1,44	1,48	9,93	12,73	5,13

0,67	2,7	1,97	0,93	1,6	1,11	1,9	5,51	7,72	1,04
1,8	1,53	2,03	0,42	1,84	1,23	1,38	3,62	3,21	1,48
1,57	3,02	2,88	1,22	2,42	2,87	1,03	3,98	5,34	1,46
1,33	3,27	2,97	0,93	2,02	1,46	0,87	2,55	9,71	1,44
0,74	3,14	2,76	1,33	3,89	2,12	0,75	5,09	5,69	1,39
1,55	1,96	3,15	1,08	2,41	1,82	1,82	2,81	2,46	1,87
1,26	1,77	2,65	1,04	1,97	1,3	1,44	2,64	4,05	4,01
1,16	1,19	1,9	1,42	2,62	1,31	1,42	3,87	3,84	1,03
0,02	3,07	1,68	0,38	2,22	2,06	0,93	5,74	10,03	0,56
0,79	2,4	3,93	1,33	2,05	1,26	0,79	4,02	6,27	0,91
1,05	1,6	1,21	0,31	1,57	1,58	1,57	2,54	4,23	0,81
1,24	1,66	2,24	0,58	1,19	1,08	1,14	2,18	2,76	0,8
0,87	2,75	2,74	0,37	1,83	1,19	1	3,65	4,35	0,87
0,7	2,96	2,33	1,45	2,71	1,84	1,53	6,9	5,18	1,05
0,92	1,88	1,88	0,91	2,6	1,79	1,73	3,4	3,54	1,1
1,27	2,33	1,43	0,97	2,15	1,45	1,09	4,06	3,99	1,9
2,4	3,99	3,82	1,86	3,05	1,87	2,14	1,29	14,69	1,23
1,33	3,35	2,88	1,07	2,29	2,07	1,41	4,33	4,1	1,46
1,32	1,58	2,36	0,59	1,98	1,16	1,61	2,49	3,01	1,11
0,7	2,76	2,06	1,19	1,8	1,36	0,47	4,06	2,41	1,54
2,66	2,9	2,53	2,46	2,79	1,64	1,84	3,11	4,71	3,92
1,54	0,78	1,81	0,55	1,96	1,35	1,99	1,18	2,53	0,67
1,96	2,85	3,33	2,36	2,48	1,51	1,77	2,92	2,99	1,34
1,51	2,3	3,97	1,31	2	1,73	2,64	4,61	3,73	1,18
1,52	2,14	2,75	0,93	1,93	1,7	1,24	1,67	2,94	0,63
1,37	3,29	2,83	1,38	2,24	1,88	0,71	4,21	4,64	2,11
1,67	1,02	2,55	0,59	1,69	1,46	2,33	1,5	3,45	1,91

Final turn texting (s)	Stand-to-texting (s)	Sit-to-stand talking (s)	Outgoing gait talking (s)	Initial turn talking (s)	Return gait talking (s)	Final turn talking (s)	Sit-to-stand talking (s)
1,07	0,92	1,3	2,68	1,35	3,03	1,59	1,61
0,82	0,84	1,28	1,37	1,18	1,63	1,39	1,09
0,9	1,22	2,7	2,08	1,77	3,62	1,41	1,09
0,65	0,28	1,83	1,62	1,04	2,52	1,06	0,93
0,3	0,55	1,41	2,25	1,06	2,39	0,24	0,48
1,28	1,26	0,97	1,59	0,62	1,23	1,39	0,4
1,54	1,43	2,01	3,78	1,95	3,88	1,49	3,1
0,97	0,47	1,55	1,91	1,27	2,53	1,14	0,63
0,92	1,44	1,25	3,22	1,55	2,28	1,3	1,89
1,78	0,8	1,67	2,03	2,15	3,33	1,4	1,45
1,08	0,17	1,1	4,9	1,32	4,29	0,94	2,65
1,47	0,18	1,62	4,04	1,74	3,87	1,33	2,65
1,64	0,86	2	1,94	1,76	4,8	1,08	1,85
1,34	0,58	1,77	3,17	1,23	4,08	1,11	1,76
1,43	1,16	1,83	1,4	0,97	1,71	1,14	0,72
0,95	1	1,8	3,03	1,79	3,46	1,04	1,58
1,03	1,23	1,42	2,93	1,74	2,75	1,31	1,38
1,22	0,98	2,23	1,45	1,34	1,88	1,24	0,83
1,24	1,52	1,87	1,37	1,11	2,71	1,09	1,14
1,55	0,47	2,09	1,46	1,36	2,05	1,25	1,61
1,11	1,13	1,44	1,61	1,17	2,15	0,77	1,14
1,45	1,09	2,15	1,71	1,51	1,97	0,98	1,54
1,23	1,28	2,83	3,31	1,24	3,14	1,33	1,26
1,32	0,99	1,51	3,02	1,45	3,79	0,94	1,38
1,27	1,27	1,52	3,45	2,23	4,52	1,51	1,6

0,98	1,29	1,28	1,82	1,34	1,77	0,95	1,17
0,9	0,26	1,57	1,85	1,01	1,84	1,2	1,57
0,96	1,45	1,88	1,73	1,3	1,65	0,94	1,49
1,23	1,71	1,59	2	1,54	1,98	1,15	1,31
1,1	1,63	2,2	2,46	1,35	3,01	1,41	0,34
1,39	0,49	1,62	1,54	1,22	1,81	0,24	3,85
1,01	1,47	0,86	3,38	1,48	3,05	0,86	1,83
1,02	0,88	1,36	1,53	1,37	1,6	1,22	1,16
1,37	1,54	2,01	2,15	1,72	1,67	1,41	1,68
0,78	1,52	1,67	2,75	1,54	3,89	1,06	1,01
1,4	1,27	1,94	2,07	1,24	2,02	1,21	1,85
1,32	2,03	1,99	3,69	1,39	4,63	1,32	1,64
1,12	1,03	1,81	3,12	1,98	4,09	1,08	0,95
1,64	0,29	1,52	2,53	1,37	3,05	0,99	2,04
1,03	1,76	2,14	1,63	1,47	1,92	1,09	1,4
0,9	1,22	2,7	2,08	1,77	3,62	1,41	1,09
0,65	0,28	1,83	1,62	1,04	2,52	1,06	0,93
0,97	0,47	1,55	1,91	1,27	2,53	1,14	0,63
0,97	0,47	1,55	1,91	1,27	2,53	1,14	0,63
0,3	0,55	1,41	2,52	1,06	2,39	0,24	0,48
1,07	0,92	1,3	2,68	1,35	3,03	1,59	1,61
0,82	0,84	2,28	1,37	1,18	1,63	0,39	1,09
1,5	1	1,8	2,5	1,6	3	1,4	1,2
1,2	1,1	1,9	2,6	1,7	3,2	1,3	1,3
1,25	1,05	1,85	2,55	1,65	3,05	1,35	1,25
0,65	0,28	1,83	1,62	1,04	2,52	1,06	0,93
1,34	0,58	1,77	3,17	1,23	3,08	1,11	1,76
0,65	0,28	1,83	1,62	1,04	2,52	1,06	0,93
1,22	0,98	2,23	1,45	1,34	1,88	1,24	0,83

0,96	1,45	1,88	1,73	1,3	1,65	0,94	1,49
1,02	0,88	1,36	1,53	1,37	1,6	1,22	1,16
0,96	1,45	1,88	1,73	1,3	1,65	0,94	1,49
0,97	0,47	1,55	1,91	1,27	2,53	1,14	0,63
1	0,68	1,08	2,01	1,18	2,4	1,7	1,41
1,01	1,47	0,86	3,38	1,48	3,05	0,86	1,83
1,55	0,47	2,09	1,46	1,36	2,05	1,25	1,61
1,26	1,63	1,92	1,51	1,38	2,73	1,22	1,2
1,59	7,72	1,79	5,87	2,88	0,81	1,98	3,64
1,56	5,41	0,71	2,4	1,9	1,5	1,78	2,1
2,1	3,92	1,82	4,58	2,3	1,7	1,96	3,79
2,71	4,45	1,9	4,8	2,98	2,15	2,27	3,98
2,27	5,34	1,68	3,54	2,49	1,39	2,93	4,28
2,01	1,54	2,01	3,78	3,88	3,1	1,95	1,49
1,42	1,07	1,3	2,68	3,03	1,61	1,35	1,59
1,31	1,89	2,91	3,25	4,72	2,25	2,19	1,56
2,43	1,44	0,97	4,48	3,54	1,31	1,69	1,26
2,15	1,82	2,03	3,47	3,95	3,28	2,04	1,73
1,53	1,17	1,45	2,07	2	2,02	1,6	1,67
1,55	1,63	1,92	3,1	4,77	0,74	2,19	1,38
3,44	1,66	1,82	5,15	7,69	2,84	3,02	2,61
3,09	2,46	2,24	4,5	8,1	4,67	2,71	2,8
2,54	1,28	1,2	6,85	7,3	4,66	1,73	2,17
2,24	1,85	3,61	6,7	11,39	2,12	2,34	1,06
2,88	1,67	1,48	7,53	10,07	4,07	2,69	1,64
0,54	1,38	1,74	4,76	4,18	0,89	1,55	1,42
2,2	1,9	1,06	7,47	8,92	2,8	1,9	1,96
2,77	3,44	2,6	12,47	21,61	3,74	6,91	3,26
1,78	1,53	1,94	4,13	4,53	2,09	1,74	1,54

2,09	2,81	2,78	5,56	6,08	2,38	2,47	2,97
2,42	0,94	1,59	6,54	8,04	1,57	1,99	1,26
3,14	2,14	2,63	5,76	6,51	5,05	2,17	3
6,16	1,14	1,08	5,86	5,8	0,84	2,25	0,27
1,47	1,51	1,35	2,27	3,01	1,11	1,47	0,98
2,3	2	1,03	10,71	9,36	1,63	1,32	1,54
5,24	1,65	2,11	1,68	6,45	3,09	3,17	1,83
1,83	1,37	1,95	3,71	4,24	3,28	2,1	1,54
2,51	1,8	1,65	5,08	5,17	1,62	1,84	1,58
3,58	2,12	1,37	2,19	4,42	2,42	3,91	2,39
3,35	1,69	2,13	7,92	7,91	2,56	2,66	2,38
6,51	1,48	1,64	10,06	10,12	3,02	0,98	2,16
2,31	1,84	2,38	3,8	4,88	3,26	1,78	1,5
2,42	0,94	1,59	6,54	8,04	1,57	1,99	1,26
1,93	1,59	1,79	5,87	3,64	0,81	2,88	1,98
2,07	1,83	1,78	2,35	3,02	1,9	1,67	2,15
2,58	2,27	2,09	2,49	4,74	1,6	1,92	2,03
8,59	2,92	0,9	5,69	5,39	1,02	2,71	2,28
5,27	2,78	0,7	4,79	8,63	1,85	4,02	2,99
3,95	2,08	1,84	2,84	4,72	2,26	4,27	2,04
3,12	2,71	1,95	1,6	6,72	1,74	3,65	2,32
1,63	1,18	1,75	2	3,06	0,99	2,03	1,7
0,98	2,25	0,49	3,82	5,01	1,8	3,84	2,03
4,3	1,3	0,66	3,47	6,62	2,07	2,71	2,41
1,55	2	1,36	1,34	2,15	1,54	1,5	1,7
2,15	1,44	1,23	1,51	1,93	0,6	2,37	1,34
3,4	1,58	0,73	4,57	5,54	1,28	2,57	2,28
2,88	1,3	1,8	2,46	3,66	2,26	2,6	2,2
4,24	1,97	1,69	1,53	4,41	1,37	3,65	1,34

2,47	1,26	1,65	3,31	3,99	1,87	1,88	2,27
0,48	2,6	1,09	5,83	10,71	1,19	1,11	2,59
3,43	2,25	1,99	2,68	3,68	1,2	3,32	2,55
2,22	2,13	1,15	1,59	2,37	1,08	1,91	1,76
2,16	1,35	0,85	2,65	1,65	1,58	2,08	2,17
2,2	2,2	1,74	2,63	2,74	3,29	3,82	1,76
3,15	1,45	1,12	1,77	4,9	0,94	2,61	1,35
3,62	3,22	1,74	3,62	6,01	2,96	3,87	2,12
2,29	2,04	5,11	3,47	4,61	0,41	2,53	1,13
2,11	1,95	0,88	2,33	4,31	0,38	3,16	2,42
2,76	1,55	1,34	5,36	4,68	2,63	2,32	2,65
3,13	1,44	1,9	2,22	2,65	3,55	3,25	2,41
